# Distributed Leadership and Teachers’ Social and Emotional Learning Practices in Chinese Schools: A Relational Coordination Perspective Using TALIS 2024 Data

**DOI:** 10.3390/bs16040523

**Published:** 2026-04-01

**Authors:** Mengting Qian, Xianjun Yu, Jingyi Yang, Chunshun Yan

**Affiliations:** 1 Faculty of Education, East China Normal University, 3663 North Zhongshan Road, Putuo District, Shanghai 200062, China; mengtingqian.edu@gmail.com (M.Q.); chunshun.yan@mail.utoronto.ca (C.Y.); 2 Berkeley School of Education, University of California, Berkeley, 2121 Berkeley Way, Berkeley, CA 94720, USA; 3 Music Education, School of Music, College of Fine Arts, Boston University, 855 Commonwealth Avenue, Boston, MA 02215, USA; 4 School of Education and Communication, Jingmen Vocational College, Jingmen 448000, China; yangjingyijm@163.com; 5 Ontario Institute for Studies in Education, University of Toronto, Toronto, ON M5S 1V6, Canada

**Keywords:** distributed leadership, social–emotional learning, teacher collaboration, teacher empathy, Relational Coordination Theory, TALIS 2024

## Abstract

Although social and emotional learning (SEL) has become increasingly prominent in global education policy, how school leadership shapes teachers’ SEL teaching practices remains insufficiently understood, particularly in non-Western contexts. Grounded in Relational Coordination Theory, this study examined the mechanisms through which distributed leadership influences teachers’ SEL teaching practices, with teacher collaboration and teacher empathy specified as sequential mediators. Using data from 1299 teachers in the China sample of the Teaching and Learning International Survey (TALIS) 2024, the study tested a sequential mediation model through bootstrap-based mediation analysis (PROCESS Model 6). Confirmatory factor analysis supported the measurement model, and discriminant validity was established through nested model comparisons. The results showed that distributed leadership was both directly and indirectly associated with SEL teaching practices, with the indirect associations accounting for 62.77% of the total association. Teacher empathy emerged as the most influential mediator (45.74%), followed by the sequential mediation pathway through teacher collaboration and teacher empathy (12.16%), whereas the specific indirect effect of teacher collaboration was comparatively small (4.87%). These findings extend Relational Coordination Theory to the educational context by illustrating how organizational leadership practices may be linked to classroom-level SEL teaching behaviors through relational processes and teachers’ emotional capacities. The study underscores that advancing SEL implementation may require not only structural reform in the distribution of leadership but also the systematic cultivation of two interrelated mechanisms: professional collaboration and teacher empathy.

## 1. Introduction

Social and emotional learning (SEL) has become a central issue in global educational reform, aiming to cultivate students’ competencies in self-awareness, emotion management, social awareness, interpersonal skills, and responsible decision-making (Collaborative for Academic, Social, and Emotional Learning [CASEL], 2020). Empirical evidence has shown that SEL programs can significantly improve students’ academic achievement, mental health, and prosocial behavior (Cipriano et al., 2023; Greenberg, 2023). However, the effective implementation of SEL depends heavily on teachers’ classroom practices, as teachers’ instructional behaviors directly shape the quality of students’ social–emotional development (Jennings & Greenberg, 2009; Schonert-Reichl, 2017).

In the Chinese educational context, the education system has long been oriented toward academic achievement under the influence of the Confucian cultural tradition, and SEL has not been incorporated into the mainstream curriculum (Zong et al., 2024). In recent years, however, SEL has gradually entered the policy agenda with the advancement of the “double reduction” policy and the growing salience of student mental health concerns (Li & Hesketh, 2024). Nevertheless, teachers in this context generally lack the professional knowledge and skills needed to integrate SEL into instruction (Zong et al., 2024). This situation highlights the urgent need to examine the key factors that shape teachers’ SEL teaching practices. It should be noted that, in the present study, SEL teaching practices are defined specifically as the frequency with which teachers engage in concrete classroom instructional behaviors intended to foster students’ social–emotional competencies, including helping students understand and manage emotions, develop empathy, and build healthy relationships. This construct is distinct from related but broader concepts, such as classroom climate, emotional support, or the traditional Chinese notion of moral education. Although moral education in China and SEL overlap to some extent at the philosophical level—particularly in their shared concern with character development and social responsibility—the SEL teaching practices measured in the Teaching and Learning International Survey (TALIS) 2024 focus on instructional behaviors targeting specific social–emotional skills (Organisation for Economic Co-operation and Development [OECD], 2025), rather than the broader moral-ethical socialization process embedded in Chinese educational philosophy.

School leadership is a core organizational factor shaping teachers’ instructional behavior. Distributed leadership theory emphasizes that leadership practice emerges through interactions among multiple actors and organizational structures (Spillane et al., 2004; Harris & Jones, 2019; G. Wang & Bai, 2025). Empirical studies have shown that distributed leadership promotes teachers’ professional collaboration and self-efficacy (Hsieh et al., 2024; J. Liu et al., 2023), and positively predicts student-centered instructional practices (Veletić et al., 2024). However, how it is associated with teachers’ SEL teaching practices has not yet been systematically examined. Moreover, although distributed leadership is generally associated with positive educational outcomes, research has also pointed to its boundary conditions and potential limitations. In particular, in cultural systems characterized by high power distance, hierarchical authority structures may constrain the extent to which leadership can be genuinely shared (Tian et al., 2016). Understanding how distributed leadership operates in such cultural contexts is therefore especially important.

Relational Coordination Theory provides an analytical framework for understanding the mechanisms underlying these effects. This theory posits that effective coordination depends on the relational network among organizational members, including relational dimensions (shared goals, shared knowledge, and mutual respect) and communication dimensions (frequent, timely, and accurate communication) (Gittell, 2002, 2015; Bolton et al., 2021). Research has shown that high levels of relational coordination enhance organizational performance and member well-being (Bolton et al., 2021; Spitzer et al., 2023). The present study adopts Relational Coordination Theory rather than alternative frameworks, such as transformational leadership theory or organizational learning theory, because it uniquely captures the dual mechanisms through which leadership practice permeates organizational processes: structural mechanisms (communication) and relational mechanisms (trust and respect). It is therefore particularly well suited to examining the collaboration and empathy pathways hypothesized in this study.

Drawing on Relational Coordination Theory, this study identifies two mediating variables: teacher collaboration and teacher empathy. Within this framework, we focus on teacher empathy rather than broader constructs such as social–emotional competence or emotion regulation for two reasons. First, empathy represents the individual-level psychological manifestation of mutual respect, a core relational dimension in Relational Coordination Theory, and thus serves as a concrete expression of high-quality working relationships (Gittell, 2016). Second, TALIS 2024 provides a validated teacher empathy scale that directly measures teachers’ capacity to perceive and respond to students’ emotional states in classroom contexts. Theoretically, this is the most proximal teacher-level factor predicting SEL teaching practices (Jennings & Greenberg, 2009). Teacher collaboration reflects professional working relationships among teachers built on shared goals and mutual respect (Gittell, 2015). Distributed leadership enhances teacher collaboration through a shared vision and participatory decision-making (Khasawneh et al., 2023; Y. Wang & Fan, 2025), which in turn promotes improvements in instructional practice (Hudson, 2024). Teacher empathy is a critical competence for implementing SEL, enabling teachers to recognize and respond to students’ emotional needs (Jennings & Min, 2023; Poulou & Garner, 2024). Teachers with higher levels of empathy are more likely to adopt emotionally supportive instructional strategies (Aldrup et al., 2024; Graziano et al., 2024). Teacher collaboration may further enhance empathy by fostering professional dialogue around SEL, thereby generating a sequential mediating effect, but this possibility has not yet been empirically tested.

Relevant research conducted in Western contexts provides preliminary evidence for the proposed pathways. For example, Llorent and Núñez-Flores (2023), in a study of teachers across different educational stages in Spain, found that teachers demonstrated relatively high levels of social–emotional and moral competence, with significant differences by gender and teaching experience. Gimbert et al. (2023), in a systematic review of research on educators’ SEL competence, emphasized that teachers’ own social–emotional competence is a prerequisite for effective SEL implementation. Collie et al. (2025) further noted in a review that teachers’ perceived social–emotional competence is a key psychological mechanism linking school organizational conditions to instructional behavior. However, the applicability of these findings to the Chinese educational context remains to be verified.

In summary, the existing literature has several limitations. First, there is a lack of an integrated framework, grounded in Relational Coordination Theory, for explaining how distributed leadership is associated with teachers’ SEL teaching practices. Second, the proposed sequential mediating effect has not yet been tested. Third, the applicability of these relationships in the Chinese context remains unclear. The timing of the present study is especially appropriate because TALIS 2024 is the first survey cycle to include dedicated measures of both teacher empathy and SEL teaching practices, providing an unprecedented opportunity to examine these relationships using internationally standardized instruments.

Based on Relational Coordination Theory and using TALIS 2024 data from China, this study examines the mechanisms through which distributed leadership influences teachers’ SEL teaching practices. Specifically, the study addresses the following research questions: (1) Does distributed leadership directly predict teachers’ SEL teaching practices? (2) Do teacher collaboration and teacher empathy serve as mediators? (3) Is there a sequential mediating effect? This study aims to deepen the theoretical understanding of how school leadership shapes teacher practice and to provide practical implications for advancing SEL in Chinese schools.

## 2. Literature Review and Hypotheses

### 2.1. Relational Coordination Theory

Relational Coordination Theory was proposed by Gittell to explain how organizational members achieve effective task integration through high-quality communication and relationships (Gittell, 2002). The theory comprises two mutually reinforcing dimensions: a relational dimension (shared goals, shared knowledge, and mutual respect) and a communication dimension (communication that is frequent, timely, accurate, and oriented toward problem-solving) (Gittell et al., 2020). Research has shown that these elements jointly promote coordinated interactions among organizational members, thereby enhancing organizational performance and individual work behavior (Bolton et al., 2021). In recent years, Relational Coordination Theory has been widely applied in fields that depend heavily on cross-role collaboration, such as healthcare, aviation, and education. Studies have further shown that when leaders adopt empowering and participative leadership approaches, the level of relational coordination among members can be significantly strengthened (Gittell, 2016; Van Staalduinen et al., 2023).

**Figure 1:**
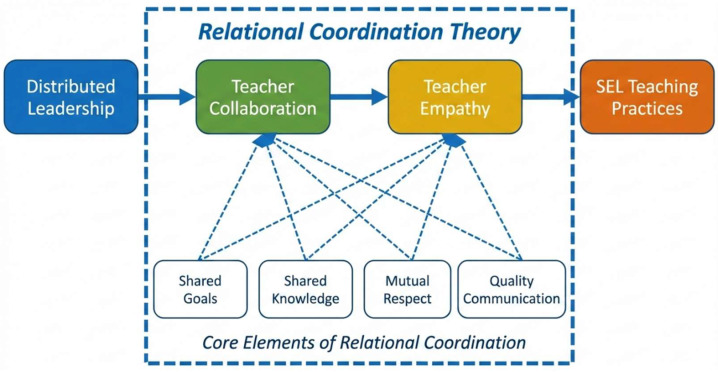
Theoretical Framework Based on Relational Coordination Theory.

Introducing Relational Coordination Theory into the school context is of substantial theoretical value. As illustrated in Figure 1, distributed leadership emphasizes the sharing of authority and participation in decision-making and can therefore be understood as an antecedent that activates relational coordination among teachers (S. Liu & Hallinger, 2021). Within the framework of Relational Coordination Theory, shared goals and shared knowledge encourage teachers to form collaborative professional communities, whereas mutual respect and high-quality communication help cultivate teacher empathy, a key social–emotional competence involving the capacity to understand and respond to others’ emotions (Hadar et al., 2020). Together, these four core elements underpin the two mediating variables in this study—teacher collaboration and teacher empathy—and ultimately facilitate teachers’ implementation of SEL teaching practices in the classroom (Collie, 2020). Accordingly, Relational Coordination Theory provides a strong theoretical foundation for understanding the sequential pathway linking distributed leadership, teacher collaboration, teacher empathy, and SEL teaching practices.

### 2.2. The Relational Ecology of Chinese Schools: Contextualizing SEL and Leadership

Chinese school organizations exhibit a distinctive relational ecology, which provides an important contextual foundation for understanding the relationship between distributed leadership and SEL teaching practices. Unlike Western schools, which tend to emphasize individual autonomy and flatter decision-making structures, Chinese schools are deeply rooted in the Confucian cultural tradition and are characterized by both high power distance and collectivism (Truong, 2013). This cultural foundation has shaped a distinctive pattern of “relational authority,” in which the exercise of principal leadership is often embedded in interpersonal trust, moral influence, and collective harmony (Zhu & Caliskan, 2021).

Against this backdrop, the practice of distributed leadership in Chinese schools reflects indigenous features that integrate both rational and relational considerations. Research has shown that principals in this context tend to share leadership responsibilities by establishing institutionalized collaborative mechanisms, such as teaching-research groups and mentoring systems. These arrangements reflect the Confucian collectivist emphasis on shared learning rather than individual competition (Y. Liu et al., 2021). At the same time, the high power distance characteristic of Chinese schools may result in relatively limited direct interaction between principals and frontline teachers, such that distributed leadership often operates indirectly through middle-level teacher leaders (Zheng et al., 2021). This cultural pattern suggests that the effects of distributed leadership may differ from those observed in Western contexts. In high power distance systems, the sharing of leadership may depend more on institutionalized collaborative structures than on individual-level empowerment, which may in turn shape distinctive pathways for the development of teacher collaboration and empathy.

With regard to SEL, Chinese schools have long regarded moral education as a central educational goal, yet systematic SEL teaching practices remain at a developmental stage (Chen & Yu, 2022). Importantly, teachers in these schools face a distinctive tension in implementing SEL. On the one hand, pressure associated with the National College Entrance Examination and large class sizes constrain opportunities for teachers to conduct experiential social–emotional activities. On the other hand, the professional ethic of “teaching and nurturing the whole child” assigns teachers a moral responsibility to attend to students’ holistic development (S. Liu & Hallinger, 2021). In addition, the implementation of the “double reduction” policy has created new opportunities for schools to reallocate time resources and strengthen SEL practices (Xue & Li, 2023). Accordingly, examining how distributed leadership supports SEL teaching practices through the promotion of teacher collaboration and the enhancement of teacher empathy is of substantial theoretical and practical significance for understanding the relational mechanisms underlying school improvement in China.

### 2.3. Research Hypotheses

#### 2.3.1. Distributed Leadership and SEL Teaching Practices

Distributed leadership emphasizes the sharing and dispersion of leadership functions across organizational members rather than concentrating them in a single administrator (Spillane, 2005). In school settings, this leadership model provides organizational support for innovative instructional practices by granting teachers greater participation in decision-making and stronger professional autonomy (Harris & Jones, 2019). From the perspective of Relational Coordination Theory, distributed leadership essentially creates a relational network grounded in shared goals, mutual respect, and frequent communication. Such a high-quality relational coordination mechanism can effectively promote coordinated behavior among organizational members (Gittell, 2016).

As a complex form of educational innovation, SEL teaching practice requires that teachers have adequate resource support and a sense of psychological safety. Research has shown that distributed leadership can motivate teachers to experiment with new instructional approaches by fostering a climate of trust and providing opportunities for professional development (Y. Liu et al., 2021). Principals’ social–emotional competence and leadership practices also play a foundational role in shaping the overall SEL climate of schools, thereby providing the organizational support necessary for teachers to implement SEL instruction (Mahfouz et al., 2019). Recent empirical studies have further confirmed that distributed leadership significantly promotes teachers’ innovative instructional practices by enhancing teacher collaboration and strengthening teachers’ self-efficacy (Teng et al., 2024; Hsieh et al., 2024). Based on the above analysis, this study proposes the following hypothesis:

**H1.**  *Distributed leadership is significantly and positively associated with teachers’ SEL teaching practices*.

#### 2.3.2. The Mediating Effect of Teacher Collaboration

Teacher collaboration refers to the process by which teachers share knowledge, exchange experiences, and coordinate instructional actions in professional practice through activities such as joint lesson planning, peer observation, reflective dialogue, and collaborative problem-solving (Vangrieken et al., 2015). This concept goes beyond simple information exchange and highlights deep professional interaction among teachers based on shared goals and interdependence (Duyar et al., 2013). In contemporary research on school organizations, teacher collaboration is regarded as a key mechanism linking school leadership to improvements in instructional practice.

Relational Coordination Theory provides a powerful analytical framework for understanding how distributed leadership may influence instructional practice through teacher collaboration. The theory emphasizes that high-quality working relationships depend on three core relational dimensions—shared goals, shared knowledge, and mutual respect—which are reinforced through communication that is frequent, timely, accurate, and oriented toward problem-solving (Gittell, 2002). In school settings, distributed leadership provides the structural foundation for building this kind of high-quality coordinated relationship among teachers through mechanisms of shared authority and distributed responsibility (Harris & Jones, 2019). More specifically, when leadership functions are distributed across school members, teachers have greater opportunities to participate in collective decision-making processes, thereby facilitating the development of shared goals and the flow of professional knowledge (Wenner & Campbell, 2017). Empirical evidence has shown that distributed leadership can significantly promote teacher collaboration (Y. Liu et al., 2021).

According to the reciprocal logic of Relational Coordination Theory, the trust and shared understanding developed in collaborative processes are translated into higher-quality instructional outcomes (Gittell et al., 2010). In the specific context of SEL teaching, collaborative practice enables teachers not only to acquire professional knowledge about SEL instruction but also to develop, through mutual support, the emotional skills and practical wisdom needed to implement such instruction effectively (Schonert-Reichl, 2017). Recent research has further confirmed that teacher collaboration is a key pathway linking school leadership to instructional improvement (Meyer et al., 2022). Accordingly, this study proposes the following hypotheses:

**H2a.**  *Distributed leadership is significantly and positively associated with teacher collaboration*.

**H2b.**  *Teacher collaboration is significantly and positively associated with teachers’ SEL teaching practices*.

**H2c.**  *Teacher collaboration mediates the relationship between distributed leadership and teachers’ SEL teaching practices*.

#### 2.3.3. The Mediating Effect of Teacher Empathy

Teacher empathy refers to teachers’ ability to understand, perceive, and respond to students’ emotional states, encompassing two dimensions: cognitive empathy (understanding others’ perspectives) and affective empathy (experiencing others’ emotions) (Goroshit & Hen, 2016). In the context of social–emotional learning instruction, teacher empathy is regarded as a core competence for the effective implementation of SEL curricula (Jennings & Greenberg, 2009).

From the perspective of Relational Coordination Theory, distributed leadership can significantly enhance teacher empathy by fostering an organizational climate characterized by shared goals and mutual respect. When leadership responsibilities and authority are distributed across multiple actors in a school, teachers participate more frequently in decision-making processes and assume leadership roles. This shift in role is likely to foster stronger perspective-taking ability and greater emotional sensitivity (Berkovich & Eyal, 2015). Empirical research has shown that distributed leadership can promote teacher professional development and the enhancement of emotional competence by cultivating a supportive school culture (Y. Liu et al., 2021). In addition, teachers with higher levels of empathy are better able to recognize students’ emotional needs and adjust their instructional strategies accordingly, thereby implementing SEL teaching practices more effectively (Aldrup et al., 2020). Recent studies have further revealed that teacher empathy is an important psychological mechanism linking school organizational factors to classroom social–emotional support behaviors (Poulou & Garner, 2024). Based on this reasoning, this study proposes the following hypotheses:

**H3a.**  *Distributed leadership is significantly and positively associated with teacher empathy*.

**H3b.**  *Teacher empathy is significantly and positively associated with teachers’ SEL teaching practices*.

**H3c.**  *Teacher empathy mediates the relationship between distributed leadership and teachers’ SEL teaching practices*.

#### 2.3.4. The Sequential Mediating Roles of Teacher Collaboration and Teacher Empathy

According to Relational Coordination Theory, high-quality coordination depends on the mutually reinforcing mechanisms of three relational dimensions: shared goals, shared knowledge, and mutual respect (Bolton et al., 2021). In school organizational contexts, teacher collaboration, as a form of deep professional interaction, provides a critical relational foundation for the development of teacher empathy. When teachers continuously engage in perspective exchange, experience sharing, and collective reflection during collaborative processes, their perspective-taking ability and emotional understanding can be significantly strengthened (Martinsone & Žydžiūnaite, 2023). Research has shown that frequent professional collaboration helps teachers move beyond the limits of individual cognition and cultivate greater sensitivity to students’ emotional needs through in-depth dialogue with colleagues (Khasawneh et al., 2023; Vangrieken et al., 2015).

Furthermore, enhanced teacher empathy provides both an emotional and a cognitive foundation for SEL teaching practices. Teachers with stronger empathy are better able to identify students’ social–emotional needs and translate this understanding into supportive classroom interactions and emotionally responsive instructional strategies (Jennings & Greenberg, 2009). Research has confirmed that teachers’ social–emotional competence not only influences classroom management and the quality of teacher-student relationships, but also directly determines the fidelity and effectiveness of SEL program implementation (Schonert-Reichl, 2017; Oberle et al., 2020). Therefore, distributed leadership may foster a collaborative culture that promotes professional interaction among teachers; collaborative practice may then cultivate teacher empathy through relational processes, ultimately advancing high-quality SEL teaching practices. Accordingly, this study proposes the following hypotheses:

**H4a.**  *Teacher collaboration is significantly and positively associated with teacher empathy*.

**H4b.**  *Teacher collaboration and teacher empathy play a sequential mediating role in the relationship between distributed leadership and teachers’ SEL teaching practices*.

**Figure 2:**
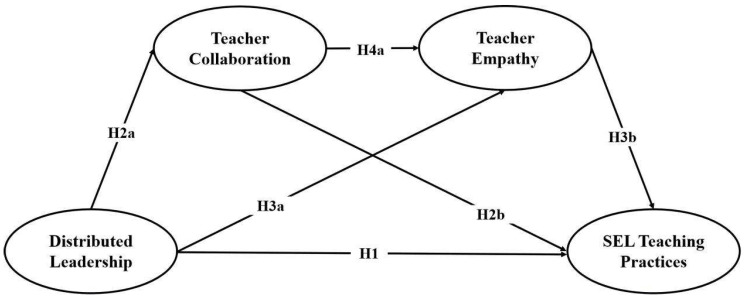
Research Framework.

Based on the above assumptions, this study developed a research framework, as shown in Figure 2.

## 3. Methods

### 3.1. Participants

The data used in this study were drawn from the Teaching and Learning International Survey (TALIS 2024), conducted in 2024 by the Organisation for Economic Co-operation and Development (OECD). TALIS 2024 is a large-scale international comparative study of teachers and principals in primary and secondary education that is designed to systematically examine educators’ working conditions, professional development, and instructional practices. The TALIS 2024 China sample primarily covers lower secondary school teachers.

This study used the Chinese teacher sample as the unit of analysis. The original dataset included 4078 teachers. To ensure the robustness of the findings, listwise deletion was used to screen the sample. Cases were excluded if they had missing values on the core study variables (i.e., all measurement items for distributed leadership, teacher collaboration, teacher empathy, and SEL teaching practices) or on the control variables (gender, age, employment status, and target class size). After this procedure, the final analytic sample consisted of 1299 valid cases, representing a retention rate of 31.9%.

To assess the potential selection bias introduced by sample screening, the demographic characteristics of the retained sample were compared with those of the full sample. The results showed that the retained sample and the full sample were similar in their distributions of gender (73.0% female in the retained sample vs. 74.0% female in the full sample), age group (in the retained sample, 19.8% were aged 29 or younger, 30.7% were aged 30–39, 29.9% were aged 40–49, 19.2% were aged 50–59, and 0.4% were aged 60 or older; the corresponding proportions in the full sample were 19.0%, 30.2%, 30.4%, 20.1%, and 0.2%, respectively), employment status (91.4% full-time in the retained sample vs. 90.2% full-time in the full sample), and target class size (67.8% of the retained sample vs. 68.2% of the full sample taught classes with 35 or more students). These results suggest that listwise deletion did not introduce substantial demographic selection bias. In addition, outliers in the four core variables were examined through exploratory data analysis using boxplots and stem-and-leaf plots. All observed values fell within the plausible ranges of their respective scales, and no extreme outliers were identified that warranted removal.

The demographic characteristics of the sample are presented in Table 1. In terms of gender composition, female teachers constituted the majority, with 948 teachers (73.0%), whereas 351 teachers were male (27.0%). With respect to age distribution, the sample was composed primarily of early- and mid-career teachers. The largest age group was 30–39 years (399 teachers, 30.7%), followed by 40–49 years (388 teachers, 29.9%). In terms of employment status, full-time teachers accounted for the vast majority of the sample (1187 teachers, 91.4%). Regarding target class size, more than two-thirds of the teachers (881 teachers, 67.8%) taught classes with 35 or more students.

### 3.2. Measures

#### 3.2.1. Distributed Leadership

In this study, distributed leadership was treated as the independent variable and measured using relevant items from the distributed leadership scale in the TALIS 2024 teacher questionnaire. Based on theoretical considerations and exploratory factor analysis, four items with relatively high factor loadings and strong theoretical alignment were retained. All items were rated on a four-point Likert scale (1 = “Strongly disagree,” 2 = “Disagree,” 3 = “Agree,” 4 = “Strongly agree”), with higher scores indicating higher levels of distributed leadership perceived by teachers in their schools.

The four items were: TT4G64G (“This school encourages staff to lead new initiatives”), TT4G64I (“Teachers take leadership roles in promoting a professional learning community”), TT4G64J (“Teachers initiate and lead collaborative activities”), and TT4G64K (“Teachers lead their professional growth and development activities”).

These four items were specified as observed indicators of a single latent construct, distributed leadership, in a confirmatory factor analysis (CFA). The results showed that the standardized factor loadings ranged from 0.809 to 0.949, all well above the recommended threshold of 0.50. The average variance extracted (AVE) was 0.831, and the composite reliability (CR) was 0.951, indicating good convergent validity. Because the scale was unidimensional and included only four items, the model was relatively parsimonious and demonstrated excellent fit: *χ*^2^/df = 0.771, GFI = 0.999, RMR = 0.001, AGFI = 0.997, and SRMR = 0.002. The major fit indices all met recommended criteria, indicating good structural validity and suggesting that the scale can effectively measure the level of distributed leadership in schools.

#### 3.2.2. Teacher Collaboration

Teacher collaboration was treated as a mediating variable and measured using the teacher collaboration scale from the TALIS 2024 teacher questionnaire. Based on theoretical analysis and exploratory factor analysis, six items with relatively high factor loadings and consistency with the theoretical structure were retained. The scale used a six-point Likert response format (1 = “Never,” 2 = “Once a year or less,” 3 = “2–4 times a year,” 4 = “5–10 times a year,” 5 = “1–3 times a month,” 6 = “Once a week or more”), with higher scores indicating more frequent participation in collaborative activities.

The six items were: TT4G26B (“Observe other teachers’ classes and provide feedback”), TT4G26D (“Exchange teaching materials with colleagues”), TT4G26E (“Engage in discussions about learning development”), TT4G26F (“Work with other teachers to ensure common standards”), TT4G26G (“Take part in collaborative professional learning”), and TT4G26H (“Collaborate with parents or guardians”).

Scores were calculated as the mean of the six items. These six items were specified as observed indicators of a single latent construct, teacher collaboration, in a confirmatory factor analysis. The scale showed good internal reliability, with an overall Cronbach’s α of 0.866. The CFA results indicated an acceptable level of model fit: GFI = 0.962, CFI = 0.961, NFI = 0.958, NNFI = 0.935, TLI = 0.935, and SRMR = 0.037, suggesting that the scale adequately captures the frequency of teachers’ participation in collaborative activities.

#### 3.2.3. Teacher Empathy

Teacher empathy was treated as the second mediator in the sequential mediation model and measured using relevant items from the teacher empathy scale in the TALIS 2024 teacher questionnaire. This scale consisted of five items rated on a four-point Likert scale (1 = “Never or almost never,” 2 = “Sometimes,” 3 = “Often,” 4 = “Always”), with higher scores indicating stronger empathy demonstrated by teachers in teaching their target class.

The five items were: TT4G60A (“Be aware of my students’ feelings”), TT4G60B (“Show warmth to my students”), TT4G60C (“Care about the problems of my students”), TT4G60D (“Be empathetic towards my students”), and TT4G60E (“Care about the social and emotional problems of my students”).

Scores were calculated as the mean of the five items. These five items were specified as observed indicators of a single latent construct, teacher empathy, in a confirmatory factor analysis (CFA). Reliability testing showed good internal consistency, with an overall Cronbach’s α of 0.949. The CFA results indicated acceptable structural validity: GFI = 0.957, RMR = 0.007, CFI = 0.980, NFI = 0.979, NNFI = 0.960, and TLI = 0.960. The major fit indices all met recommended standards, suggesting that the scale can effectively measure teachers’ level of empathy.

#### 3.2.4. SEL Teaching Practices

SEL teaching practices were treated as the dependent variable and measured using relevant items from the development of students’ social–emotional skills scale in the TALIS 2024 teacher questionnaire. This scale consisted of six items rated on a four-point Likert scale (1 = “Never or almost never,” 2 = “Sometimes,” 3 = “Often,” 4 = “Always”), with higher scores indicating more frequent implementation of social–emotional learning-related instructional practices in the target class.

The six items were: TT4G61A (“Understanding their own emotions, thoughts, or behaviour”), TT4G61B (“Managing their own emotions, thoughts, or behaviour”), TT4G61C (“Understanding the perspectives of others”), TT4G61D (“Empathising with others”), TT4G61E (“Establishing and maintaining healthy relationships with others”), and TT4G61F (“Making caring and constructive choices about their personal actions”).

Scores were calculated as the mean of the six items. These six items were specified as observed indicators of a single latent construct, SEL teaching practices, in a confirmatory factor analysis (CFA). Reliability testing showed good internal consistency, with an overall Cronbach’s α of 0.957. The CFA results indicated acceptable structural validity: GFI = 0.968, RMR = 0.005, CFI = 0.986, NFI = 0.985, NNFI = 0.977, and TLI = 0.977. The major fit indices all met recommended criteria, suggesting that the scale can effectively measure teachers’ level of SEL teaching practices.

#### 3.2.5. Control Variables

Previous research has shown that demographic characteristics may influence teachers’ SEL teaching practices. In this study, gender, age, employment status, and target class size were included in the model as control variables.

### 3.3. Data Analysis

Given that TALIS 2024 employed a stratified cluster sampling design in which teachers were nested within schools, this study first calculated intraclass correlation coefficients (ICCs) for the four core variables to assess school-level clustering effects. The results showed that the ICC was 0.103 for distributed leadership, 0.066 for teacher collaboration, 0.018 for teacher empathy, and approximately 0.000 for SEL teaching practices. Except for distributed leadership, the ICCs for the other three variables were all below the commonly used threshold of 0.10, suggesting that overall school-level clustering effects were limited. The ICC for distributed leadership was slightly above 0.10, which is theoretically reasonable given that leadership practice is itself an organizational characteristic at the school level (Stapleton et al., 2016). Taken together, because the central focus of this study was on individual teacher-level mediating mechanisms and because most variables exhibited low ICCs, the use of single-level mediation analysis was considered appropriate.

With respect to the analytic strategy, this study employed the PROCESS macro (Hayes, 2017) rather than a full structural equation modeling (SEM) approach for three main reasons. First, PROCESS Model 6 provides a well-established procedure for testing multiple sequential mediation effects and allows the simultaneous estimation and comparison of the relative contributions of different indirect pathways (Hayes, 2017). Second, the primary purpose of this study was to examine mediation mechanisms using observed variables rather than structural relations among latent variables; regression-based path analysis in PROCESS was therefore sufficient for addressing the research questions. Third, the CFA results had already provided adequate evidence for the measurement quality of the scales, thereby justifying the use of mean scores for the observed variables as inputs for the regression analyses. At the same time, the PROCESS approach separates the measurement and structural models and cannot incorporate sampling weights. The implications of this analytic choice are discussed in Section 7.

Common method bias testing, descriptive statistical analyses, and correlation analyses were conducted using SPSS 27.0. The relationships among distributed leadership, teacher collaboration, teacher empathy, and SEL teaching practices were then examined using Model 6 of the PROCESS macro (Hayes, 2017). Specifically, a sequential mediation model was specified, with distributed leadership as the independent variable, SEL teaching practices as the dependent variable, and teacher collaboration and teacher empathy as the sequential mediators, while controlling for gender, age, employment status, and target class size. A bias-corrected bootstrap procedure with 5000 resamples was used to test the significance of the mediation effects and to generate 95% confidence intervals. If the lower and upper bounds of the 95% confidence interval (CI) did not include zero, the mediation effect was considered statistically significant. Multicollinearity was assessed using variance inflation factors (VIFs). All VIF values ranged from 1.200 to 1.234, well below the critical threshold of 5, indicating that multicollinearity was not a concern.

## 4. Results

### 4.1. Common Method Bias and Discriminant Validity

Because the data in this study were collected through teacher self-reports, common method bias may be a concern. To minimize this risk, the Organisation for Economic Co-operation and Development (OECD) implemented several procedural remedies during the design and administration of the TALIS 2024 questionnaire, including ensuring respondent anonymity, using varied item wording, and adopting different scale anchors. At the statistical level, this study employed two methods to assess common method bias.

First, Harman’s single-factor test showed that an unrotated exploratory factor analysis of all 21 measurement items extracted four factors with eigenvalues greater than 1, and the first factor accounted for 46.809% of the variance, which is below the critical threshold of 50%. This result suggests that a single factor did not account for the majority of the variance.

Second, as shown in Table 2, this study compared a series of nested CFA models to further examine common method bias and discriminant validity. The four-factor model, in which distributed leadership, teacher collaboration, teacher empathy, and SEL teaching practices were specified as distinct constructs, demonstrated good fit: *χ*^2^/df = 4.684, CFI = 0.974, TLI = 0.970, RMSEA = 0.053, and SRMR = 0.030. Compared with alternative models, the four-factor model fit the data significantly better than the three-factor model combining teacher empathy and SEL teaching practices (Δ*χ*^2^ = 2481.065, Δ*df* = 3, *p* < 0.001), the two-factor model (Δ*χ*^2^ = 5271.355, Δ*df* = 5, *p* < 0.001), and the one-factor model (Δ*χ*^2^ = 10178.788, Δ*df* = 6, *p* < 0.001). The one-factor model showed very poor fit (CFI = 0.585, RMSEA = 0.210), indicating that the data structure could not be explained by a single latent factor and further confirming that common method bias did not pose a serious threat to this study.

In addition, discriminant validity was assessed using the Fornell-Larcker criterion (Fornell & Larcker, 1981). As shown in Table 3, the average variance extracted (AVE) for each construct exceeded its shared variance with the other constructs (i.e., AVE > *r*^2^), supporting discriminant validity among the four constructs. In particular, the *r*^2^ between teacher empathy and SEL teaching practices was 0.582, whereas the AVE for teacher empathy was 0.791 and the AVE for SEL teaching practices was 0.795, both exceeding this shared variance value. These findings indicate that although teacher empathy and SEL teaching practices were strongly related, they remained empirically distinguishable as independent constructs at the measurement level.

To examine whether the measurement model was equivalent across gender groups, this study conducted a multigroup confirmatory factor analysis to test measurement invariance of the four-factor model across male and female teachers (Cheung & Rensvold, 2002). As shown in Table 4, the configural invariance model demonstrated good fit (CFI = 0.970, RMSEA = 0.041), and both metric invariance (ΔCFI = 0.000) and scalar invariance (ΔCFI = 0.003) satisfied the criterion of ΔCFI < 0.01. These results indicate full measurement invariance of the four-factor measurement model across male and female teachers, ensuring that the effects observed when gender was included as a control variable reflect true differences at the construct level (Cheung & Rensvold, 2002). Because the male teacher subsample was relatively small (*n* = 351), this study did not conduct gender-specific comparisons of the mediation paths. Instead, gender was included as a control variable in all regression models. Future research may further examine the moderating role of gender using larger samples.

### 4.2. Descriptive Statistics and Correlation Analysis

Table 5 presents the means, standard deviations, and correlation coefficients for all study variables. Distributed leadership (M = 3.314, SD = 0.565), teacher empathy (M = 3.414, SD = 0.577), and SEL teaching practices (M = 3.371, SD = 0.547) were all measured on four-point scales, with mean scores ranging from 3.314 to 3.414. These values suggest that teachers in the sample generally reported moderately high levels on these constructs. Teacher collaboration was measured on a six-point scale (M = 4.344, SD = 1.063), with a mean above the scale midpoint of 3.5, likewise indicating a moderately high frequency of collaborative activities. Notably, teacher empathy showed the highest relative level within its scale range, whereas teacher collaboration showed the lowest relative level among the four variables.

The correlation analysis showed that all variables were significantly and positively correlated with one another (*p* < 0.001). Specifically, distributed leadership was significantly and positively correlated with teacher collaboration (*r* = 0.341, *p* < 0.001), teacher empathy (*r* = 0.369, *p* < 0.001), and SEL teaching practices (*r* = 0.428, *p* < 0.001). Teacher collaboration was also significantly and positively correlated with teacher empathy (*r* = 0.335, *p* < 0.001) and SEL teaching practices (*r* = 0.341, *p* < 0.001). Particularly noteworthy was the strong positive correlation between teacher empathy and SEL teaching practices (*r* = 0.763, *p* < 0.001), suggesting a close association between these two constructs. Although this correlation coefficient was relatively high, the discriminant validity tests reported above indicated that the two constructs remained empirically distinguishable at the measurement level.

These preliminary findings provide initial support for the hypothesized sequential mediation model, as the correlations among distributed leadership, teacher collaboration, teacher empathy, and SEL teaching practices were all in the expected directions.

### 4.3. Hypothesis Testing

This study used Model 6 of the PROCESS macro in SPSS 27.0 to test the sequential mediating roles of teacher collaboration and teacher empathy in the relationship between distributed leadership and teachers’ SEL teaching practices. In this model, distributed leadership was specified as the independent variable, teachers’ SEL teaching practices as the dependent variable, teacher collaboration as the first mediator, and teacher empathy as the second mediator. Gender, age, employment status, and target class size were included as control variables. The regression results are presented in Table 6.

First, the direct effect was examined. The results showed that, after controlling for the mediators, distributed leadership still significantly and positively predicted teachers’ SEL teaching practices (*B* = 0.153, 95% *CI* [0.117, 0.190], *p* < 0.001), supporting Hypothesis 1. Second, the mediating pathway through teacher collaboration was tested. Distributed leadership significantly and positively predicted teacher collaboration (*B* = 0.625, 95% *CI* [0.530, 0.720], *p* < 0.001), and teacher collaboration also significantly and positively predicted teachers’ SEL teaching practices (*B* = 0.031, 95% *CI* [0.012, 0.051], *p* < 0.01). Thus, Hypotheses 2a and 2b were supported. Third, the mediating pathway through teacher empathy was examined. Distributed leadership significantly and positively predicted teacher empathy (*B* = 0.295, 95% *CI* [0.242, 0.347], *p* < 0.001), and teacher empathy also significantly and positively predicted teachers’ SEL teaching practices (*B* = 0.639, 95% *CI* [0.603, 0.676], *p* < 0.001). Therefore, Hypotheses 3a and 3b were supported. Finally, the precondition for the sequential mediation pathway was tested. Teacher collaboration significantly and positively predicted teacher empathy (*B* = 0.125, 95% *CI* [0.097, 0.154], *p* < 0.001), supporting Hypothesis 4a.

With respect to the control variables, gender showed significant effects in multiple models. Female teachers scored higher than male teachers on teacher empathy (B = −0.172, *p* < 0.001) and SEL teaching practices (*B* = −0.055, *p* < 0.05). Because gender was coded as 1 = female and 2 = male, the negative coefficients indicate higher scores for female teachers. This pattern is consistent with previous findings on gender differences among teachers (Graziano et al., 2024). Age also significantly and positively predicted teacher empathy (*B* = 0.054, *p* < 0.001) and SEL teaching practices (*B* = 0.023, *p* < 0.05), suggesting that the accumulation of teaching experience may contribute to the development of teachers’ social–emotional competence. Employment status and target class size were not significant in the final model after all mediators were included.

Given that gender showed significant effects across multiple models, this study conducted additional gender-stratified analyses. Specifically, PROCESS Model 6 was run separately for the male teacher subsample (*n* = 351) and the female teacher subsample (*n* = 948) to examine the robustness of the mediation mechanisms across gender groups. The results showed that, in the female teacher subsample, the pattern of the three indirect effects was consistent with that observed in the full sample: the specific indirect effect through teacher empathy was the largest (*B* = 0.193, *SE* = 0.025, 95% *CI* [0.145, 0.244]), followed by the sequential indirect effect (*B* = 0.040, *SE* = 0.008, 95% *CI* [0.025, 0.057]), whereas the specific indirect effect through teacher collaboration was the smallest (*B* = 0.018, *SE* = 0.007, 95% *CI* [0.004, 0.033]). In the male teacher subsample, the total indirect effect was also significant (*B* = 0.268, *SE* = 0.053, 95% *CI* [0.171, 0.380]), and the relative pattern of the indirect pathways was generally similar to that in the full sample. The specific indirect effect through teacher empathy was the largest (*B* = 0.169, 95% *CI* [0.082, 0.272]), followed by the sequential indirect effect (*B* = 0.076, 95% *CI* [0.046, 0.112]). However, the specific indirect effect through teacher collaboration did not reach statistical significance (*B* = 0.023, 95% *CI* [−0.005, 0.054]), which may be attributable to the relatively small male subsample and the resulting limited statistical power. Overall, these supplementary gender-stratified analyses suggest that the mediation mechanisms identified in this study were generally robust across male and female teachers.

To further examine the mediating roles of teacher collaboration (M1) and teacher empathy (M2) in the relationship between distributed leadership and teachers’ SEL teaching practices, this study used a bias-corrected percentile bootstrap procedure with 5000 resamples to test the mediation effects. The results are presented in Table 7.

The total effect of distributed leadership on teachers’ SEL teaching practices was significant (*B* = 0.411, *SE* = 0.024, 95% *CI* [0.364, 0.458]). After the two mediators were included, the direct effect remained significant (*B* = 0.153, *SE* = 0.019, 95% *CI* [0.117, 0.190]), accounting for 37.23% of the total effect. The total indirect effect was also significant (*B* = 0.258, *SE* = 0.023, 95% *CI* [0.213, 0.306]), accounting for 62.77% of the total effect.

A further decomposition of the total indirect effect showed that all three mediation pathways were significant, and the 95% confidence interval for each pathway did not include zero. Indirect Path 1 (distributed leadership → teacher collaboration → SEL teaching practices) was significant (*B* = 0.020, *SE* = 0.007, 95% *CI* [0.007, 0.033]), accounting for 4.87% of the total effect, thereby supporting Hypothesis 2c. Indirect Path 2 (distributed leadership → teacher empathy → SEL teaching practices) was also significant (*B* = 0.188, *SE* = 0.023, 95% *CI* [0.145, 0.235]), accounting for 45.74% of the total effect, thereby supporting Hypothesis 3c. Indirect Path 3 (distributed leadership → teacher collaboration → teacher empathy → SEL teaching practices), representing the sequential mediation pathway, was likewise significant (*B* = 0.050, *SE* = 0.007, 95% *CI* [0.036, 0.065]), accounting for 12.16% of the total effect, thereby supporting Hypothesis 4b.

**Figure 3:**
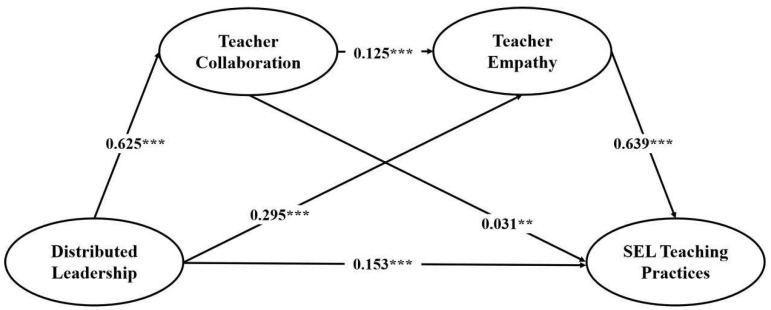
Effects of Distributed Leadership on SEL Teaching Practices. *Note.* ** *p* < 0.01, *** *p* < 0.001 (two-tailed).

Given the cross-sectional design, these mediation results should be interpreted as reflecting statistical associations rather than established causal sequences. Taken together, the association between distributed leadership and teachers’ SEL teaching practices reflects a dual pattern in which indirect effects play the primary role and the direct effect plays a secondary role. Among the three indirect pathways, the specific indirect effect through teacher empathy contributed the most (45.74%), followed by the sequential indirect effect (12.16%), whereas the specific indirect effect through teacher collaboration contributed the least (4.87%). These findings indicate that teacher empathy plays a central mediating role in the relationship between distributed leadership and SEL teaching practices. It should be noted that the proportions reported above for the indirect effects (e.g., 45.74%) reflect the relative contribution of each pathway to the total effect rather than absolute effect sizes. These proportions should therefore be interpreted alongside the absolute effect estimates: the absolute value of the specific indirect effect through teacher empathy was 0.188 (BootSE = 0.023), the absolute value of the specific indirect effect through teacher collaboration was 0.020 (BootSE = 0.007), and the absolute value of the sequential indirect effect was 0.050 (BootSE = 0.007). The relatively large proportion associated with the teacher empathy pathway is partly attributable to the strong association between teacher empathy and SEL teaching practices (*r* = 0.763), as discussed earlier in the analysis of discriminant validity. The mediation model is depicted in Figure 3.

## 5. Discussion

Grounded in Relational Coordination Theory and using TALIS 2024 data from China, this study systematically examined the mechanisms linking distributed leadership to teachers’ SEL teaching practices. The findings revealed a sequential pathway from distributed leadership through teacher collaboration and teacher empathy to SEL teaching practices, offering a new theoretical perspective on how school leadership is connected to classroom instructional practice.

First, distributed leadership was significantly and positively associated with SEL teaching practices. The results showed that distributed leadership had both a significant direct association with teachers’ SEL teaching practices, accounting for 37.23% of the total effect, and a stronger indirect association, accounting for 62.77% of the total effect. This finding is consistent with the central proposition of Relational Coordination Theory that high-quality organizational coordination depends on relational networks characterized by shared goals, shared knowledge, and mutual respect among members (Gittell, 2016; Bolton et al., 2021). Through mechanisms of shared authority and distributed responsibility, distributed leadership may promote teachers’ shared understanding of the value of SEL (Spillane, 2005; Harris & Jones, 2019). Notably, the indirect association was substantially larger than the direct association, suggesting that distributed leadership may be linked to SEL teaching primarily by activating teacher-level mediating mechanisms rather than through simple top-down administrative influence. This finding is in line with recent research indicating that the association between school leadership and instructional practice is often indirect and teacher-mediated (Buyukgoze et al., 2024). At the same time, an alternative explanation is also possible: teachers who frequently engage in SEL teaching practices may be more likely to perceive leadership support in their schools or to participate more actively in distributed leadership processes. Future longitudinal research is needed to clarify the directionality of this relationship.

Second, teacher collaboration mediated the relationship between distributed leadership and SEL teaching practices, although its specific indirect effect was relatively small, accounting for 4.87% of the total effect. From the perspective of Relational Coordination Theory, teacher collaboration reflects the features of communication among organizational members that are frequent, timely, and oriented toward problem-solving (Gittell et al., 2020). By establishing a shared vision and participatory decision-making mechanisms, distributed leadership provides structural support for deep professional interaction among teachers (Ma & Marion, 2024; Zheng et al., 2021). However, the relatively small specific indirect effect of teacher collaboration may reflect the distinctive features of the study context. In high power distance cultures, teacher collaboration may focus more on exchanges related to instructional techniques and less on in-depth dialogue about the principles and practices of social–emotional instruction (Y. Liu et al., 2021; Qian & Yan, 2026). It should be noted, however, that this cultural interpretation remains speculative, as the present data do not allow direct examination of differences in the content of collaboration. In addition, SEL has not yet developed into a fully institutionalized professional discourse within Chinese schools (Zong et al., 2024), which may further limit the direct translation of collaborative activities into SEL teaching practices. It is also important to note that TALIS measures the frequency rather than the quality of collaboration. Teachers may participate in collaborative activities frequently, but whether these activities are sufficiently substantive or explicitly focused on SEL-related issues remains an open question for future research.

Third, teacher empathy showed the strongest mediating effect, accounting for 45.74% of the total effect, and thus played a central role in the overall mechanism. Relational Coordination Theory emphasizes mutual respect as one of the foundational dimensions of high-quality working relationships (Gittell, 2016), and teacher empathy can be understood as the individual-level psychological manifestation of mutual respect. Teachers with higher levels of empathy are better able to recognize and respond to students’ emotional needs and may therefore be more capable of translating organizational leadership support into concrete social–emotional teaching behaviors in the classroom (Aldrup et al., 2024; Poulou & Garner, 2024). This finding echoes the central proposition of the prosocial classroom model that teachers’ social–emotional competence is a key prerequisite for high-quality SEL instruction (Jennings & Greenberg, 2009; Jennings & Min, 2023). The prominent role of teacher empathy suggests that efforts to improve school leadership in ways that effectively promote SEL teaching practices may need to focus not only on organizational structures, but also on the cultivation of teachers’ emotional competence. At the same time, given the high correlation between teacher empathy and SEL teaching practices (*r* = 0.763), caution is needed in interpreting this pathway, as the conceptual proximity between the two constructs may have inflated the observed mediation effect. This issue is discussed further in Section 7.

It is also worth noting that the final model explained a relatively large proportion of variance in SEL teaching practices (R^2^ = 0.614), which is uncommon in self-report survey research and therefore warrants careful consideration. In the present study, this result is likely attributable to two factors. First, from a theoretical perspective, teacher empathy and SEL teaching practices are closely related conceptually. Empathy is itself a core prerequisite for effective SEL instruction (Jennings & Greenberg, 2009), so a high degree of covariation between the two in teachers’ everyday instructional behavior is theoretically plausible. Second, from a methodological perspective, both constructs were measured through teacher self-reports, using the same four-point response scale and at the same time point. Shared method variance may therefore have inflated the strength of their association to some extent. Although the earlier discriminant validity tests, including nested CFA model comparisons and the Fornell-Larcker criterion, indicated that the two are empirically distinguishable constructs, it remains important to acknowledge that common method variance and conceptual proximity between predictors and outcomes likely contributed to the relatively high R^2^, as discussed in Section 7. Future research should further test the robustness of this association using multisource data, such as classroom observations to assess SEL teaching practices and student ratings to assess teacher empathy.

Finally, the sequential mediating effect of teacher collaboration and teacher empathy was supported, accounting for 12.16% of the total effect. This pathway reveals the dynamic linkage between organizational factors and individual capacities within the framework of Relational Coordination Theory. The collaborative culture fostered by distributed leadership may provide teachers with sustained opportunities for professional dialogue, while the exchange of perspectives, sharing of experience, and collective reflection embedded in collaborative processes may promote the development of teachers’ perspective-taking ability and emotional sensitivity (Martinsone & Žydžiūnaite, 2023; Khasawneh et al., 2023). This progressive pathway—from organizational structure to interpersonal interaction to individual capacity—reflects the core mechanism emphasized by Relational Coordination Theory, namely the mutually reinforcing relationship between the relational and communication dimensions of coordination (Bolton et al., 2021). The presence of this sequential mediation effect suggests that, in promoting SEL teaching practices, schools need not only to build collaborative platforms but also to attend to the quality of collaborative activities so that they can serve as effective vehicles for cultivating teacher empathy.

## 6. Theoretical and Practical Implications

At the theoretical level, this study situates the relationship between distributed leadership and SEL teaching practices within the dual relational-communication framework of Relational Coordination Theory, thereby addressing a key gap in the literature, namely, the lack of a mechanism-based explanation for how leadership is linked to classroom SEL practice. By introducing teacher collaboration and teacher empathy and constructing a sequential mediation model, this study proposes a progressive pathway from organizational structure (distributed leadership) to relational process (collaboration), then to emotional capacity (empathy), and ultimately to instructional behavior (SEL teaching practices). Beyond confirming previously suggested relationships, the present study identifies the differential roles of the relational and communication dimensions of coordination: teacher empathy (relational dimension) contributed substantially more than teacher collaboration (communication dimension), suggesting that interpersonal relationship quality may matter more than interaction frequency for SEL implementation. At the same time, the present study offers only a preliminary extension of Relational Coordination Theory, primarily demonstrating the applicability of this framework to the school context rather than making a substantive revision of the theory itself. Future research could further examine how specific dimensions of Relational Coordination Theory, such as shared goals, shared knowledge, and mutual respect, differentially shape teacher practice.

At the practical level, the findings generate differentiated implications for multiple stakeholder groups. For school leaders and administrators, promoting the implementation of SEL should not rely solely on administrative advocacy. Instead, schools should establish routine, problem-solving-oriented collaborative structures, such as joint lesson planning, peer observation, and case-based discussion, so that leadership responsibilities are meaningfully aligned with SEL goals. Given that teacher empathy accounted for a more substantial share of the association between leadership and SEL practice, teacher development efforts should explicitly incorporate empathy into school-based professional learning and teaching-research group activities, for example, through contextualized training and reflective exercises focused on recognizing students’ emotions and engaging in responsive communication. For policymakers, school support conditions that foster teacher collaboration and the development of emotional competence could be incorporated into indicators for SEL implementation, thereby promoting a systematic improvement pathway that attends to both structural empowerment and relational development. For teacher educators, both preservice preparation and in-service training should place greater emphasis on the systematic cultivation of social–emotional competence, especially empathy, so that teachers enter the classroom already equipped with the core capacities needed to implement SEL instruction effectively.

## 7. Limitations and Future Research

This study has several limitations, which should be taken into account when interpreting the findings.

First, this study was based on cross-sectional data from the TALIS 2024 China sample. Although such data allow for the examination of structural relationships among variables and the testing of a sequential mediation model, they do not support causal inference. All associations reported in this study should be understood as correlational, and the term “mediation” as used here refers to statistical indirect associations rather than established causal mechanisms. In particular, the ordering of teacher collaboration before teacher empathy in the sequential mediation model was derived from theoretical assumptions rather than from temporal evidence. As a result, reverse causality—for example, teachers with higher levels of empathy may be more likely to participate actively in collaborative activities—or reciprocal relationships cannot be ruled out. Future research should employ longitudinal designs, cross-lagged panel models, or quasi-experimental approaches to verify the temporal ordering and causal direction of these relationships.

Second, all variables were derived from teacher self-reports and may therefore have been influenced by social desirability bias. Although the tests of common method bias and discriminant validity suggested that this issue was not severe, reliance on a single data source remains an important limitation. In particular, the measurement of SEL teaching practices captured only teachers’ self-reported frequency of these behaviors and could not reflect the quality or depth of classroom implementation. Future studies should integrate multisource data, such as classroom observations, student feedback, and peer or principal ratings, to strengthen measurement validity and to distinguish between the frequency and quality of SEL implementation.

Third, the PROCESS macro used in this study separates the measurement and structural models rather than estimating them simultaneously. A full structural equation modeling (SEM) approach would have had the advantage of accounting for measurement error in the latent constructs, potentially yielding more accurate parameter estimates and allowing direct tests of model fit at the structural level. In addition, the PROCESS macro does not support the application of TALIS sampling weights (TCHWGT). Because TALIS employs a complex, stratified two-stage cluster sampling design intended to ensure national representativeness, the omission of weights means that the results cannot be interpreted as representative of the broader Chinese lower secondary teacher population. Furthermore, this study relied on listwise deletion to handle missing data, which reduced the sample from 4078 to 1299 teachers (a retention rate of approximately 32%). Although the retained and excluded samples were demographically similar, teachers who did not respond to key construct items may differ systematically from respondents, and the substantial sample reduction may have affected parameter precision, particularly for smaller indirect effects. Multiple imputation or full information maximum likelihood (FIML) estimation would have been preferable but was not compatible with the PROCESS macro. Moreover, although the ICC analysis indicated that school-level clustering was generally limited, the ICC for distributed leadership (0.103) indicates a non-negligible school-level component. Ignoring this clustering may lead to underestimated standard errors for paths involving distributed leadership. Future research should adopt multilevel structural equation modeling (MSEM) to simultaneously separate school-level and teacher-level effects, incorporate sampling weights, and address missing data more rigorously.

Fourth, the TALIS 2024 teacher questionnaire did not collect information on teachers’ subject specialization or school level taught, and this prevented the present study from examining differences in SEL teaching practices across subject areas or educational stages. Given that teachers at different educational levels, such as primary and secondary education, may differ substantially in their working environments and patterns of teacher-student interaction, future research should incorporate school-level information to explore these boundary conditions.

Fifth, although the model emphasized the core mediating mechanisms, it may still have omitted important school-level and individual-level moderating factors, such as school culture and resource allocation, institutionalized teaching-research arrangements, teacher workload, and occupational burnout. These factors may shape the relationships identified in this study. In addition, because the sample consisted of lower secondary school teachers in China, caution is needed in generalizing the findings across regions, educational levels, and cultural contexts. Future research should conduct cross-cultural comparisons to test the replicability of these findings across different cultural and educational systems and to examine the role of potential moderators such as psychological safety and trust climate.

Sixth, the strong correlation between teacher empathy and SEL teaching practices (r = 0.763) warrants careful interpretive consideration. Although discriminant validity was supported through nested CFA model comparisons and the Fornell–Larcker criterion, both constructs involve teachers’ responsiveness to students’ emotional needs, raising the possibility that they partially capture the same underlying construct. This conceptual overlap may have inflated the mediation pathway through teacher empathy. Relatedly, the final model explained a notably high proportion of variance in SEL teaching practices (R^2^ = 0.614), which is likely attributable in part to this construct’s proximity as well as to shared method variance arising from single-source, same-format self-report measurement. Future research should employ differentiated measurement strategies—such as observer ratings for SEL teaching practices and self-reports for empathy—to reduce shared method and construct overlap and to provide more conservative estimates of the mediation effects.

## Figures and Tables

**Table 1:** Demographic Characteristics of the Study Sample.

Characteristics	Sample (*N* = 1299)	
	*n*	%
**Gender**		
Female	948	73.0%
Male	351	27.0%
**Age (years)**		
≤29	257	19.8%
30–39	399	30.7%
40–49	388	29.9%
50–59	250	19.2%
≥60	5	0.4%
**Employment Status**		
Full-time	1187	91.4%
Part-time	112	8.6%
**Target Class Size**		
<15	5	0.4%
15–24	98	7.5%
25–34	315	24.2%
≥35	881	67.8%

**Table 2:** Confirmatory Factor Analysis: Model Comparison for Discriminant Validity.

Model	*χ* ^2^	*df*	*χ* ^2^ */df*	CFI	TLI	RMSEA	SRMR	Δχ^2^ (Δdf)
Four-factor model (baseline)	857.221	183	4.684	0.974	0.970	0.053	0.030	—
Three-factor model (TE + SEL merged)	3338.286	186	17.948	0.879	0.864	0.114	0.050	2481.065(3) ***
Two-factor model (TE + SEL + TC merged)	6128.576	188	32.599	0.773	0.746	0.156	0.126	5271.355(5) ***
One-factor model	11,036.009	189	58.392	0.585	0.539	0.210	0.160	10,178.788(6) ***

*Note.* TE = Teacher Empathy; SEL = SEL Teaching Practices; TC = Teacher Collaboration. *** *p* < 0.001.

**Table 3:** Discriminant Validity: AVE and Squared Correlations.

	DL	TC	TE	SEL
AVE	0.831	0.529	0.791	0.795
DL	—			
TC (*r*^2^)	0.116	—		
TE (*r*^2^)	0.136	0.112	—	
SEL (*r*^2^)	0.183	0.116	0.582	—

*Note.* DL = Distributed Leadership; TC = Teacher Collaboration; TE = Teacher Empathy; SEL = SEL Teaching Practices. AVE > *r*^2^ indicates adequate discriminant validity.

**Table 4:** Measurement Invariance Testing Across Gender.

Model	*χ* ^2^	*df*	CFI	RMSEA	ΔCFI
Configural invariance (Unconstrained)	1149.587	366	0.970	0.041	—
Metric invariance (Measurement weights)	1180.347	383	0.970	0.040	0.000
Scalar invariance (Measurement intercepts)	1270.567	404	0.967	0.041	0.003

*Note.* ΔCFI < 0.01 indicates invariance holds at each level.

**Table 5:** Descriptive Statistics and Correlation Analysis (*N* = 1299).

Variable	*M*	*SD*	1	2	3	4
1 = Distributed Leadership	3.314	0.565	1			
2 = Teacher Collaboration	4.344	1.063	0.341 ***	1		
3 = Teacher Empathy	3.414	0.577	0.369 ***	0.335 ***	1	
4 = SEL Teaching Practices	3.371	0.547	0.428 ***	0.341 ***	0.763 ***	1

*Note. M* = mean, *SD* = standard deviation. *** *p* < 0.001 (two-tailed).

**Table 6:** Regression Analysis between Variables.

Outcome Variable	Independent Variable	*R* ^2^	Adjusted *R*^2^	*F*	*B*	*SE*	*β*	*t*	*p*	Bias-Corrected 95% *CI*	
										Lower	Upper
SEL Teaching Practices		0.217	0.214	71.772 ***							
	Gender				−0.189 ***	0.031	−0.153	−6.172	0.000	−0.249	−0.129
	Age				0.044 **	0.013	0.082	3.318	0.001	0.018	0.070
	Employment Status				−0.110 *	0.048	−0.056	−2.275	0.023	−0.204	−0.015
	Target Class Size				−0.026	0.021	−0.030	−1.220	0.223	−0.067	0.016
	Distributed Leadership				0.411 ***	0.024	0.424	17.226	0.000	0.364	0.458
Teacher Collaboration		0.143	0.139	43.017 ***							
	Gender				−0.210 **	0.062	−0.088	−3.380	0.001	−0.332	−0.088
	Age				−0.118 ***	0.027	−0.114	−4.410	0.000	−0.171	−0.066
	Employment Status				−0.218 *	0.098	−0.057	−2.218	0.027	−0.410	−0.025
	Target Class Size				−0.025	0.043	−0.015	−0.585	0.559	−0.109	0.059
	Distributed Leadership				0.625 ***	0.049	0.332	12.883	0.000	0.530	0.720
Teacher Empathy		0.215	0.211	58.959 ***							
	Gender				−0.172 ***	0.032	−0.133	−5.311	0.000	−0.236	−0.109
	Age				0.054 ***	0.014	0.096	3.823	0.000	0.026	0.081
	Employment Status				−0.087	0.051	−0.042	−1.702	0.089	−0.187	0.013
	Target Class Size				−0.035	0.022	−0.039	−1.591	0.112	−0.079	0.008
	Distributed Leadership				0.295 ***	0.027	0.289	11.007	0.000	0.242	0.347
	Teacher Collaboration				0.125 ***	0.014	0.231	8.668	0.000	0.097	0.154
SEL Teaching Practices		0.614	0.612	292.894 ***							
	Gender				−0.055 *	0.022	−0.045	−2.531	0.011	−0.098	−0.012
	Age				0.023 *	0.009	0.043	2.409	0.016	0.004	0.041
	Employment Status				−0.030	0.034	−0.015	−0.882	0.378	−0.097	0.037
	Target Class Size				0.000	0.015	0.000	−0.020	0.984	−0.029	0.029
	Distributed Leadership				0.153 ***	0.019	0.158	8.219	0.000	0.117	0.190
	Teacher Collaboration				0.031 **	0.010	0.061	3.160	0.002	0.012	0.051
	Teacher Empathy				0.639 ***	0.019	0.674	34.503	0.000	0.603	0.676

*Note.* * *p* < 0.05, ** *p* < 0.01, *** *p* < 0.001 (two-tailed).

**Table 7:** Total, Direct, and Indirect Effects of Distributed Leadership on SEL Teaching Practices.

Path	*B*	*SE*	Bias-Corrected 95% *CI*		Ratio
			Lower	Upper	
Total effect	0.411	0.024	0.364	0.458	100%
Direct effect	0.153	0.019	0.117	0.190	37.23%
Total indirect effect	0.258	0.023	0.213	0.306	62.77%
Indirect path 1: DL→TC→SEL	0.020	0.007	0.007	0.033	4.87%
Indirect path 2: DL→TE→SEL	0.188	0.023	0.145	0.235	45.74%
Indirect path 3: DL→TC→TE→SEL	0.050	0.007	0.036	0.065	12.16%

*Note.* DL = Distributed Leadership; TC = Teacher Collaboration; TE = Teacher Empathy; SEL = SEL Teaching Practices. Bootstrap sample = 5000.

## Data Availability

This study used OECD TALIS 2024 data, publicly available at http://www.oecd.org/education/talis/ (accessed on 8 October 2025).
